# Interplays between drugs and the gut microbiome

**DOI:** 10.1093/gastro/goac009

**Published:** 2022-04-08

**Authors:** Yating Wan, Tao Zuo

**Affiliations:** 1 Department of Medicine and Therapeutics, Faculty of Medicine, The Chinese University of Hong Kong, Hong Kong, P. R. China; 2 Guangdong Institute of Gastroenterology, The Sixth Affiliated Hospital of Sun Yat-sen University, Guangzhou, Guangdong, P. R. China; 3 Center for Gut Microbiota Research, The Sixth Affiliated Hospital of Sun Yat-sen University, Guangzhou, Guangdong, P. R. China

**Keywords:** gut microbiota, microbiome, drug, metabolism, efficacy

## Abstract

The gut microbiota is considered a key ‘metabolic organ’. Its metabolic activities play essential roles complementary to the host metabolic functions. The interplays between gut microbes and commonly used non-antibiotic drugs have garnered substantial attention over the years. Drugs can reshape the gut microorganism communities and, vice versa, the diverse gut microbes can affect drug efficacy by altering the bioavailability and bioactivity of drugs. The metabolism of drugs by gut microbial action or by microbiota–host cometabolism can transform the drugs into various metabolites. Secondary metabolites produced from the gut microbial metabolism of drugs contribute to both the therapeutic benefits and the side effects. In view of the significant effect of the gut microbiota on drug efficiency and clinical outcomes, it is pivotal to explore the interactions between drugs and gut microbiota underlying medical treatments. In this review, we describe and summarize the complex bidirectional interplays between gut microbes and drugs. We also illustrate the gut-microbiota profile altered by non-antibiotic drugs, the impacts and consequences of microbial alteration, and the biochemical mechanism of microbes impacting drug effectiveness. Understanding how the gut microbes interact with drugs and influence the therapeutic efficacy will help in discovering diverse novel avenues of regulating the gut microbes to improve the therapeutic effects and clinical outcomes of a drug in precision.

## Introduction

A gigantic amount of microorganisms, including bacteria, viruses, fungi, and archaea, are co-residing in the human gastrointestinal (GI) tract, living in a commensal relationship with humans [1]. They are involved in the regulation of a multitude of host metabolic aspects, contributing to the digestion of foods, signaling transmission, and immunity development [2–5].

Intestinal microbiota can affect host physiology and disease pathogenesis through its structural component lipopolysaccharide or secreted metabolites that transmit via blood circulation [6]. Alterations of the gut microbiome are associated with several physical conditions, including gastrointestinal dysfunctions, cardiovascular diseases, metabolic disorders, and even diseases associated with psychiatric abnormalities [7–10]. In addition, the interest in associations between gut microbes and non-antibiotic drugs usage has been growing in recent years. Importantly, oral administration of medication is a convenient and widely used medication administration route by which drug digestion and absorption are mostly carried out in the GI tract. Therefore, gut microbes are considered a key participator in drug metabolism. Research has shown that the gut microbiota was able to influence the effect of >30 approved drugs [11] and that >200 approved compounds can inhibit the growth of at least one bacterium [12].

**Figure 1.**
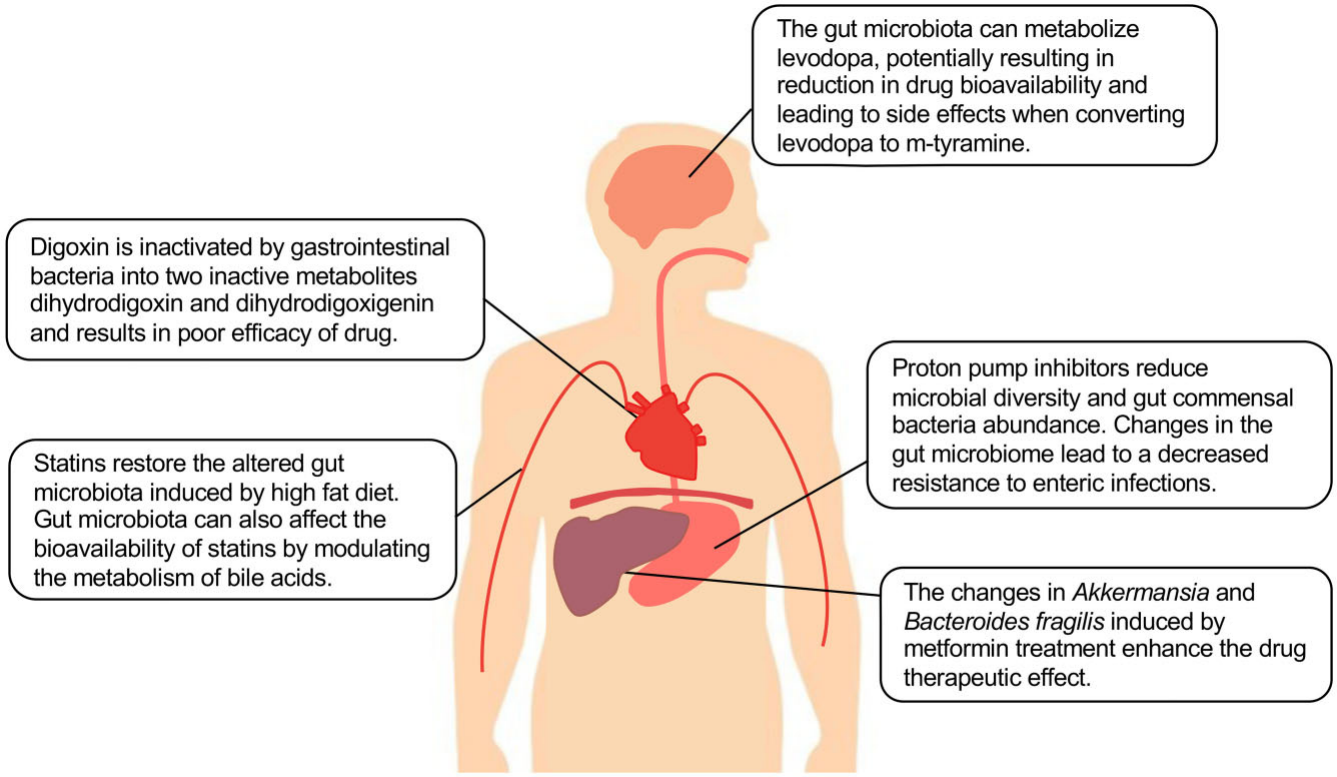
Overview of interactions between non-antibiotic drugs and the gut microbes.

The clinical outcomes are various after the administration of the same medication in patients. Some individuals experience significant improvements whereas others see less of a benefit or even no improvements at all [13]. Considering that the human gut microbiota is highly individualized, it leads one to speculate that gut microbiota is one of the primary variates determining the effectiveness of the drugs. Knowledge of how human gut microbiota affects drug pharmacokinetics and pharmacodynamics over the last decades has made big strides and further advance our understanding of inter-individual variations in drug efficacy and adverse effects. In this review, we discuss the complex bidirectional interactions between commonly used non-antibiotic drugs and the gut microbiome, and describe the microbial impact on drug efficacy and safety underlying biochemical mechanisms. Elegant examples of drug–microbiome interactions are provided, including the drugs metformin and proton-pump inhibitors (PPIs) that modulate microbiome composition and functions [14, 15] (Figure 1). Meanwhile, the gut microbiota can influence the treatment effectiveness of a specific drug by impacting its concentration and bioactivity, such as in the cases of statin, levodopa, and digoxin (Figure 1). Understanding the relationship between the gut microbiota and non-antibiotic drugs could provide us with useful and valuable instructions for assessing current drug-administration routes and the development of precision medicine.

## Metformin alters the gut microbiome of type 2 diabetes patients, contributing to its therapeutic effects

Type 2 diabetes is a disorder of blood glucose regulation (hyperglycemia) primarily arising from insulin resistance [16]. Treatment involves drug therapy and lifestyle intervention, where the blood-glucose-lowering compound metformin is the most viable drug option for type 2 diabetes [17]. The primary metabolic effect of metformin is inhibiting gluconeogenesis in the liver [18]. Compared with oral dosing, intravenously administered metformin does not enhance blood glucose regulation [19], underscoring a potentially critical role of the GI tract and its inhabiting microbes in this process. The fact that microbial mediation has beneficial effects on glucose metabolism under metformin treatment was revealed in both animals and humans [20, 21]. In humans, the changes in the abundance of the phyla Firmicutes and Bacteroidetes were significantly correlated with changes in serum cholic acid and its conjugates, which were elevated after metformin withdrawal [21]. It suggests that such microbiome changes may contribute to the therapeutic effect of metformin. In a murine study, higher abundance of the mucin-degrading bacteria *Akkermansia* and more mucin-producing goblet cells were observed in metformin-treated, high-fat diet (HFD)-fed mice than in HFD-fed mice without metformin treatment [20]. Increased *Akkermansia muciniphila* significantly enhanced glucose tolerance and attenuated adipose tissue inflammation in HFD-fed mice [20]. Meanwhile, another murine study revealed that the reduction in *Bacteroides fragilis* abundance and its selective bile salt hydrolase (BSH) activities both contributed to the improvement in glucose tolerance induced by metformin [22]. These data indicate that *A. muciniphila* and *B. fragilis* may enhance the therapeutic effect of metformin through immune-modulation and microbial metabolic processes.

Alterations in the gut microbiome composition and functions induced by metformin treatment were demonstrated in both mice [20] and humans [22, 23]. Moreover, metformin-induced changes in the gut microbiota are also diet-dependent [20]. HFD induced an increase in the abundance of Firmicutes and a decrease in the abundance of Bacteroidetes and Verrucomicrobia in mice compared with those fed with a normal chow diet (NCD) [20]. Metformin administration led to a profound shift in the fecal microbial profile of HFD-fed mice, where the abundances of Firmicutes, Bacteroidetes, and Verrucomicrobia were largely changed [20]. In contrast, there were no significant differences in these phyla between NCD-fed mice with and without metformin treatment. The abundances of 29 bacterial genera were differentiated between mice treated with and without metformin, which suggests that metformin is associated with changes in these taxa [20]. The relative abundance of *Akkermansia*, *Parabacteroides*, *Odoribacter*, *Alistipes*, *Blautia*, and *Lactonifactor* was altered by HFD, but metformin restored the abundances of these taxa to levels comparable with that in NCD-fed mice [20]. In particular, *Akkermansia* was considered a dominant contributor to the gut-microbiome difference between HFD-fed mice with and without metformin treatment [20].

**Figure 2.**
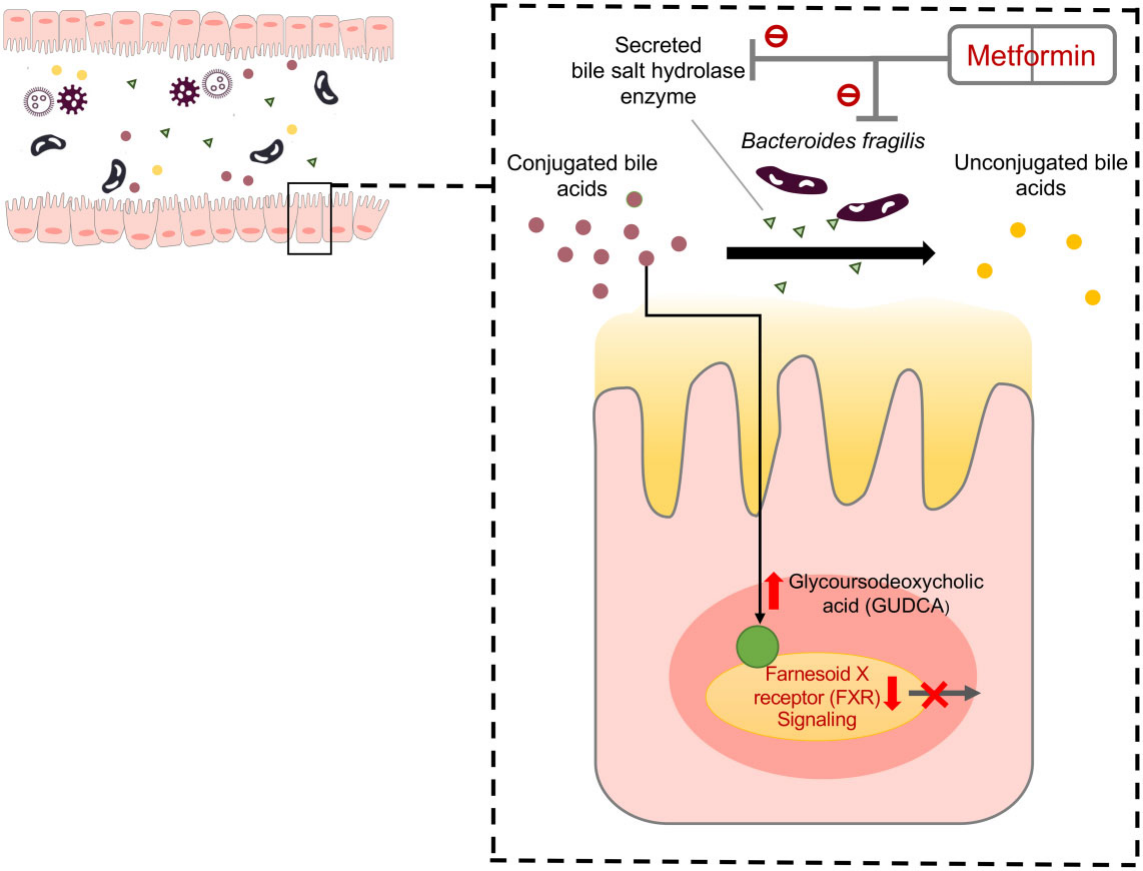
Regulation of gut microbiota–bile acid–farnesoid X receptor (FXR) axis to improve type 2 diabetes. Metformin reduces the abundance of *B. fragilis* and inhibits bile salt hydrolase (BSH) activity. These changes can further increase levels of GUDCA (endogenous FXR antagonists) and suppress the FXR signaling. It shows beneficial effects on metabolic diseases dependent on intestinal FXR inhibition.

This finding was verified in humans, further supporting that *A. muciniphila* was the most increased taxon and the mere species that was increased in abundance in response to metformin [23]. Associations of metformin usage and the abundance of *Bifidobacterium adolescentis* [24], *Escherichia coli* [25] and *A. muciniphila* [20] were also observed. *In vitro*, the growth of *B. adolescentis* and *A. muciniphila* was promoted by metformin in pure cultures yet no promotion effect was seen for *E. coli* [23]. To further investigate the functionality changes in the gut microbiome in response to metformin exposure, fecal samples from humans were cultured in a gut-simulator system with the presence of metformin, followed by whole-genome shotgun sequencing at both the DNA and RNA levels [23]. The results showed that microbiome pathways involved in butyrate and pyruvate metabolism were enriched in the metformin-treated group compared to the control group [23]. When gut microbes obtained from metformin-treated subjects (before and post-treatment) were transferred to germ-free mice fed a HFD, glucose tolerance was enhanced in mice who received fecal microbiota from post-treatment samples as compared with those who received fecal microbiota from baseline (before-treatment) samples [23]. A greater abundance of *Akkermansia* induced by metformin was demonstrated to improve the glucose tolerance in mice fed a HFD, the beneficial effect of which was similar to that of metformin-administrated mice fed a HFD [20]. *Akkermansia* had an anti-diabetic effect by reducing stromal vascular fraction inflammation in visceral adipose tissue, which was involved in the pathophysiology of insulin resistance, and by restoring the levels of Treg proportion comparable with that in metformin-treated mice fed a HFD [20]. *Akkermansia* could increase insulin signaling in HFD-fed mice through regulating immune responses [26]. In addition, metformin improved hyperglycemia via the *B. fragilis*–intestinal farnesoid X receptor (FXR) axis [22]. FXR is involved in multiple metabolic disorders by influencing glucose and lipid homeostasis [27, 28]. FXR deficiency was associated with reduced adipose tissue mass and was accompanied by glucose homeostasis and improved adipose tissue insulin sensitivity [29]. Activation of FXR induced the expression of fibroblast growth factor 15 (in mice) and 19 (in humans and other species), while metformin could suppress its activation by acting on its signaling processes [22]. Interestingly, the level of FXR expression was comparable between the mice treated with metformin plus antibiotics and mice treated with antibiotics only [22]. Hence, gut microbiota is an essential factor in intestinal FXR signaling suppression during metformin treatment. As bile acids are significant messengers in the FXR signaling, mice were gavaged with a FXR agonist taurocholic acid along with a mixture of bile acids including chenodeoxycholic acid (CDCA), glycoursodeoxycholic acid (GUDCA), and tauroursodeoxycholic acid (TUDCA) to probe their roles in the metformin–microbiome–the host axis [22]. CDCA promoted taurocholic acid-induced intestinal FXR signaling while GUDCA and TUDCA alleviated this activated signaling, suggesting that GUDCA and TUDCA are potential FXR antagonists [22]. Further, the abundance of *B. fragilis* was found to be positively correlated with FXR-activated signaling and negatively correlated with the level of potential FXR antagonists, including GUDCA and TUDCA, in stool and serum samples [22]. Interestingly, metformin could inhibit the growth of *B. fragilis* and downregulated BSH gene expression in *B. fragilis* to increase GUDCA levels to inhibit FXR signaling [22] (Figure 2). Collectively, metformin has an anti-diabetic effect through mediating the level and activities of *B. fragilis* and influencing the FXR signaling in the gut. *Bacteroidesfragilis* administration markedly abrogated the effect of metformin, characterized by impaired glucose tolerance and insulin sensitivity [22]. All these studies in rodents and humans suggest that gut microbial changes induced by metformin might enhance its therapeutic effect. The *B. fragilis*–GUDCA–FXR axis represents a critical inner working mechanism for the effect of metformin–microbiome cooperation on the host.

## Alterations in gut microbiota by cholesterol-lowering drugs and its possible beneficial roles in drug metabolism

The relationship between rosuvastatin (a commonly used cholesterol-lowering drug of the statin class) and the gut microbiota was extrapolated from a clinical trial [30]. Blood lipid levels of 64 patients with hyperlipidemia showed a significant reduction after the rosuvastatin treatment [30]. Interestingly, blood lipid levels of half of patients slumped to normal levels, presented by a reduction in the low-density lipoprotein cholesterol (LDL-C) levels (58.5%) and total cholesterol levels (26.6%), while blood lipid levels of the other patients remained high after the rosuvastatin therapy [30]. On the consideration that the various gut-microbiome compositions across humans might account for the observed variations in rosuvastatin treatment efficacy, the authors found that bacteria from Firmicutes and Fusobacteria had a negative correlation with LDL-C levels while Cyanobacteria and Lentisphaerae had a positive correlation with LDL-C levels [30]. In a separate study conducted in mice, blood levels of triglycerides, total cholesterol, and LDL-C were reduced after a 2-week statin treatment [31]. However, the level of LDL-C from the statin-plus-antibiotics-treated group (microbes-depleted) was remarkably higher than the LDL-C level from the statin-treated group [31]. These data together suggest that the intestinal microbiome contributed to the effectiveness of the statin [31].

The genera *Bacteroides*, *Butyricimonas*, and *Mucispirillum* were found to be enriched in atorvastatin- and rosuvastatin-treated mice, where rosuvastatin was more effective than atorvastatin in restoring the altered gut microbiota induced by HFD [32]. Moreover, the abundances of these bacteria were closely correlated with host inflammation markers [32]. In addition, fecal microbiota from rosuvastatin-treated mice improved serum glucose and glucose tolerance in HFD-fed mice [32]. In humans, *Blautia* and *Anaerostipes* were positively associated with butyric acid production, whereas these bacteria were depleted in patients with acute coronary syndrome. However, acute coronary syndrome patients treated with statins had a comparable level of *Blautia* and *Anaerostipes* to that of healthy individuals [33]. These results imply that statins can restore the gut-microbiota profile that are altered in a disease setting, whereas the microbiome may work in synergism with the drug on the host.

The bioavailability of cholesterol-lowering drugs could be affected by gut microbiota via modulating the metabolism of bile acids. In humans, bile acids and statins share the same three transporters in the intestine: multidrug resistance gene 1 P-glycoprotein, multidrug resistance-associated protein 2, and organic anion-transporting polypeptide 1B1 [34, 35]. Therefore, the bile acids pool can compete with cholesterol-lowering drugs for transporters, consequently affecting the bioavailability and therapeutic efficacy of these drugs. In this process, the gut microbiota may play a role by regulating the bile acid profile. Study has revealed that increased plasma concentrations of simvastatin are positively correlated with the levels of several secondary bile acids [36]. One hypothesis is that the gut microbiota calibrates the bile acid profile impacting the competition between bile acids and statins in contending for transporters on host cells, leading to bioavailability differences in statins and its therapeutic effectiveness across different individuals due to variations in gut-microbiome configurations.

## Gut microbes reduce the bioavailability of levodopa through decarboxylation

Accumulating evidence suggests that a low diversity of gut microbiota is associated with mental-health disorders such as attention deficit hyperactivity disorder [37, 38]. When the mice got a fecal transplant from depressed patients, recipient mice developed depression-like behaviors, indicating a crucial role for microbiome in such a disease [39, 40]. Similarly, the gut microbiome can influence the effect of psychotropic drugs in modulating host mental health [41]. Parkinson’s disease (PD) is a neurological movement disorder that leads to shaking and difficulty in walking and balance, affecting >1% of the population aged >60 years [42]. The most potent medication for PD is levodopa (L-dopa), which is prescribed to alleviate motor symptoms [43]. L-dopa is absorbed into the intestine and must enter the brain so as to be converted by the aromatic amino acid decarboxylase to the neurotransmitter dopamine for it to be functional in coordinating signaling from the brain to muscles [44]. However, the GI tract is one of the major sites for dopa decarboxylation, rendering that dopamine synthesized in the periphery hardly crosses the blood–brain barrier, resulting in ineffective medication of dopa [45, 46]. One major pathway contributing to this ineffectiveness is the consecutive gut microbial dihydroxylation that converts L-dopa into non-therapeutic m-tyramine [47, 48]. Thereby, the bioavailability of L-dopa to the brain at the site of active gut microbial metabolism is one key factor determining drug efficacy. Eradication of specific bacterial clusters via antibiotics was found to improve the L-dopa therapy in both humans and mice, suggesting that drug efficacy is counteracted by certain gut bacteria [49, 50]. Moreover, peripheral dopamine may result in side effects in the GI tract as well as orthostatic hypotension and cardiac arrhythmias [51]. Overall, the gut-microbiota-mediated L-dopa metabolism may lead to poor clinical outcomes and side effects [52].

The impact of L-dopa on the gut microbiota of PD patients has also been revealed in a longitudinal study to examine the gut microbiota composition in PD patients before and after L-dopa administration. A lower relative abundance of Clostridium group IV was observed in PD patients who experienced an obvious or moderate improvement in motor impairment in response to L-dopa compared with those with a small response [53]. Alterations in the microbiome functions were also observed after treatment. A strong positive correlation between bacterial tyrosine decarboxylase (*tdc*) gene relative abundance and L-dopa treatment dose as well as the duration of disease was observed [54]. Moreover, the *tdc* gene in the fecal microbiota was significantly correlated with L-dopa dosage, suggesting bacterial *tdc* may play a role in L-dopa efficacy.

Rekdal and his colleagues [55] identified a candidate L-dopa decarboxylating enzyme, PLP-dependent tyrosine decarboxylase (pyridoxal 5′-phosphate TyrDC) that was encoded by the bacterium *Enterococcus faecalis*. TyrDC was involved in drug metabolism by catalysizing the decarboxylation of L-dopa to dopamine [55]. Another dopamine dehydroxylating strain from the species *Eggerthella lenta* was also isolated, where expression association was found between PLP-dependent decarboxylase and molybdenum cofactor-dependent dopamine dehydroxylase (Dadh) enzyme, corroborating that L-dopa could be sequentially metabolized into m-tyramine [55]. The abundance of *E. faecalis*, TyrDC, dadh, L-dopa, and dopamine metabolism in the gut microbiota from PD patients were all inter-connected, suggesting that these microorganisms and their metabolism are relevant in L-dopa conversion [55]. Hence, to make L-dopa more effective in PD patients harboring such L-dopa-utilizing bacteria, inhibitors of gut bacterial L-dopa decarboxylation are proposed to be co-administered. Considering tyrosine is a substrate preference by TyrDCs, a mimic (S)-α-fluoromethyltyrosine (AFMT) was considered and used to reduce L-dopa decarboxylation, which improved the therapeutic effect in PD mice [55]. When a mixture of L-dopa and AFMT was administered to *E. faecalis*-colonized mice, it led to an elevation in the level of L-dopa in serum [55].

## PPIs alter the composition of the gut microbiota and increase the risk of enteric bacterial infection

PPIs are commonly used to reduce stomach acid in acid-related disorders and prevent gastroduodenopathy and bleeding [56]. Although few side effects are reported in PPI users, the absolute number of PPI users presenting adverse drug responses is still high [57]. 16S rDNA-based study revealed that patients with inflammatory bowel disease and patients with irritable bowel syndrome are associated with lower diversity and changes in 20% of the gut microbiota (with relative abundances decreased or increased) after PPIs treatment [58]. While the disease itself is a confounding factor when interrogating the effect of PPIs on the gut microbiome, it precludes us from dissecting the effects of disease vs PPIs usage on the gut microbiome. To tease apart the effect of PPIs usage vs disease in affecting the gut microbiome, there have been a handful of studies investigating the effect of PPIs intake on gut-microbiome composition in healthy individuals. Two separate trials on 12 healthy volunteers [14] and 1,827 healthy twins [59] who voluntarily took PPIs showed that PPIs induced considerable changes in taxonomic composition. The former study found that taxa associated with *Clostridioides difficile* infection were significantly changed after PPIs administration [14], whereas the latter study found that the gut microbiome of PPI users was characterized by a lower abundance of commensal bacteria and lower microbial diversity compared with non-users [59]. Overall, the gut microbiome of PPI users had a reduction in the abundance of *Ruminococcaceae* and *Bifidobacteriaceae*, and an elevation in the abundance of *Enterobacteriaceae*, *Enterococcaceae*, and *Lactobacillaceae* compared with non-PPI users [14]. Changes in the microbial taxon and functionality were positively correlated with a higher drug dosage [60]. A higher resolution of taxonomic and functional pathway interrogation was facilitated by metagenomic sequencing. PPIs accounted for most of the observed associations between drugs and gut-microbiome alterations amongst the associations between 42 commonly used drugs and the gut microbiome [60]. Beyond that, PPIs were the only drug category associated with gut-microbiome compositional changes across all cohorts [60]. PPI-induced changes in the predicted microbial functions include fatty acid, lipid and L-arginine biosyntheses [60].

It is vital to recognize that microbiome changed by PPIs may actually contribute to the pathogenesis or progression of some clinical diseases. Accumulating studies found that loss of specific bacterial taxa results in weakened resistance to enteric infections, including those caused by *Clostridioidesdifficile* and by *Salmonella*, which were frequently observed in PPI users [61, 62]. The odds ratios were estimated 1.5–1.8 for infection with *C. difficile* and 2.0–4.0 for infections with other pathogens after PPIs treatment [63]. It is known that antibiotics administration induces dysbiosis of gut microbial ecology that makes one vulnerable to *C. difficile* infection afterwards; this is also true for PPIs usage [64]. In addition, one study showed that PPI treatment was associated with the clinical course in decompensated liver cirrhosis, where the gut microbiome mediated this process [65]. PPI abuse in early childhood was associated with long-term changes in the gut-microbiome development and obesity in later life [66]. Although the efficacy and safety profile of PPIs are favorable, the medical community should embark on assessing the functional consequences and impacts of the changed gut microbiome by PPIs.

## Gut flora causes inter-individual variations in the metabolism of digoxin

Orally administered cardiac glycoside drug digoxin is known to control heart problems, such as irregular heartbeats (arrhythmias) including atrial fibrillation, and it also helps to manage the symptoms of heart failure [67]. However, ∼10% of patients experienced a lower benefit of digoxin due to the substantial conversion of digoxin into relatively inactive metabolites, dihydrodigoxin and dihydrodigoxigenin [68]. One of the major sites of this conversion is again carried out in the GI tract, where metabolism of digoxin by gut microbes was well established [69, 70]. Moreover, antibiotic pretreatment reduced the secretion of dihydrodigoxin in urine and increased the level of digoxin in blood, hinting at an increase in digoxin bioavailability in the host [68]. More than 40% of ingested digoxin was converted into inactivate metabolites before its absorption in the gut; the role of gut microbiota in digoxin efficiency reveals an extra layer of information about the microbial contribution to host health that is independent of human metabolism [71].

**Figure 3.**
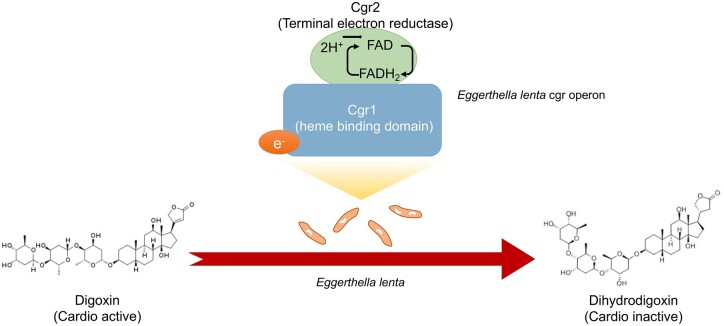
Schematic representation of digoxin-to-dihydrodigoxin conversion with the involvement of *E. lenta*. The heme binding domain of Cgr1 transfers electrons to the extra-cytoplasmic terminal electron reductase Cgr2 through heme, resulting in reduction of digoxin to dihydrodigoxin.

Research has identified some strains of digoxin-metabolizing gut bacteria, such as *E. lenta*, in individuals who can reduce the level of digoxin [72]. A two-gene cytochrome-encoding operon (namely the cardiac glycoside reductase, cgr) was also significantly upregulated in the presence of digoxin [73]. This cgr operon functions by producing a protein–Cgr1–Cgr2 complex that binds to digoxin and accounts for digoxin's consequent reduction due to the proteins that are homologous to bacterial cytochromes and are therefore potentially capable of using digoxin as an alternative electron acceptor (Figure 3). Two *E. lenta* strains that lack the operon were unable to inactivate digoxin [74]. Others found that amino acids, especially arginine, serve as the main source of nitrogen and carbon for *E. lenta* but it simultaneously inhibits digoxin inactivation [72, 73]. Therefore, a high-protein diet can help to improve the efficacy of digoxin in those patients who carry cgr + *E. lenta.*

## Conclusion and perspectives

We herein describe and summarize the relationship and interplays between non-antibiotic drugs and the gut microbiome. Clinicians and scientists should be aware that, beyond antibiotics, non-antibiotic drugs can also influence the gut-microbiome configuration and development, which may ultimately enhance or impair host health and clinical outcomes. Meanwhile, as the pharmaco-microbiomes field is coming to the surface and getting attention, a comprehensive understanding of how gut microbes metabolize/utilize/bio-transform drugs will open new potential avenues for regulating the gut microbiome to improve the efficacy of drugs and biologics. There are many clinical trials underway. For example, the clinical trial NCT04208958 (EudraCT number 2010–022394-34) is evaluating the safety and efficacy of VE800, a commensal bacterial strain formulation, in combination with Nivolumab in patients with several metastatic cancer. Another ongoing clinical trial (NCT03637803) aims to investigate the safety and efficacy of pembrolizumab in combination with a single bacterial strain *Enterococcus gallinarum* in patients with solid tumors. These trials and results would influence clinical practice in the foreseeable future.

## Authors’ Contributions

Y.T.W. conceived of the study and drafted the manuscript. T.Z. provided significant intellectual contribution and constructive advice, and revised the manuscript.

## Funding

This work was supported by the Municipal Key Research and Development Program of Guangzhou [grant number 202206010014], the National Natural Science Foundation of China [grant numbers 82172323 and 32100134], and a joint seed fund from the Sixth Affiliated Hospital of Sun Yat-sen University and Sun Yat-sen University, China.
